# Viola phlebovirus is a novel Phlebotomus fever serogroup member identified in *Lutzomyia* (*Lutzomyia*) *longipalpis* from Brazilian Pantanal

**DOI:** 10.1186/s13071-018-2985-3

**Published:** 2018-07-11

**Authors:** Michellen S. de Carvalho, Andressa Z. de Lara Pinto, Aquirya Pinheiro, Jorge S. V. Rodrigues, Fernando L. Melo, Leonardo Assis da Silva, Bergmann M. Ribeiro, Renata Dezengrini-Slhessarenko

**Affiliations:** 1 Laboratório de Virologia, Programa de Pós-Graduação em Ciências da Saúde, Universidade Federal de Mato Grosso, Cuiabá, Mato Grosso 78060-900 Brazil; 2 Laboratório de Proteção Florestal, Programa de Pós-Graduação em Ciências Florestais e Ambientais, Universidade Federal de Mato Grosso, Cuiabá, Mato Grosso 78060-900 Brazil; 3 Secretaria Estadual de Saúde de Mato Grosso, SES-MT, Laboratório de Entomologia, Cuiabá, Mato Grosso 78085-200 Brazil; 4 Departamento de Biologia Celular, Instituto de Ciências Biológicas, Universidade de Brasília, Brasília, Distrito Federal 70910-900 Brazil

**Keywords:** High throughput sequencing, Phylogeny, Viral isolation, RNA virus, Viola phlebovirus, *Bunyavirales*, *Phenuiviridae*

## Abstract

**Background:**

High throughput sequencing (HTS) boosted the discovery of novel viruses and new variants of known viruses. Here we investigated the presence of viruses in 12 pools of sand flies captured in three climatic periods in RAPELD grids at Rio Claro, Chapada dos Guimarães and at Pirizal, North Pantanal, Mato Grosso State, Midwestern Brazil by HTS, viral isolation of a putative *Phlebovirus* positive pool in Vero cells, RT-PCR and transmission electron microscopy (TEM).

**Results:**

One pool containing three *Lutzomyia* (*Lutzomyia*) *longipalpis* sand flies captured in the transitional climatic period in North Pantanal showed a tripartite genomic sequence of a putative novel *Phlebovirus* belonging to the phlebotomus fever serogroup. Phylogenetic analysis revealed this virus is closely related and share a common ancestor with phleboviruses included in the same clade: Chagres, Urucuri and Uriurana virus. RNA-dependent RNA polymerase (RdRP) presented 60%, 59% and 58% of amino-acid (aa) similarity with these phleboviruses, respectively. Similarity of Nucleoprotein and NSs protein codified by ambissense strategy of segment S was of 49% and 37%, respectively, with the proteins of the closest phlebovirus, Uriurana virus. Glycoproteins (G1, G2) and NSm protein presented 49% and 48% aa similarity with Chagres and Uriurana virus, respectively. Uriurana virus was isolated from sand flies in Brazilian Amazon and Urucuri from rodents in Utinga forest, Pará State. Chagres virus is an arbovirus responsible for outbreaks of febrile illness in Panama. This phlebovirus was isolated in Vero cells, confirmed by TEM and RT-PCR for the L segment of the virus, and named Viola phlebovirus.

**Conclusions:**

HTS, viral isolation, RT-PCR and TEM showed the presence of one virus in sand flies from North Pantanal with identity to a putative novel *Phlebovirus* from phlebotomus fever serogroup, named Viola phlebovirus.

**Electronic supplementary material:**

The online version of this article (10.1186/s13071-018-2985-3) contains supplementary material, which is available to authorized users.

## Background

Metagenomic studies based on high-throughput sequencing (HTS) have increased the discovery of new viral species and novel variants of known viruses, as well as contributed to viral ecology and evolution studies [1, 2].

The importance of arbovirus transmitted diseases dramatically increased in the past few decades, since emerging arboviruses lead to the occurrence of large viral epidemics in tropical developing regions of the world, including Brazil. Although arboviruses present worldwide distribution, viral species distribution varies among geographical regions. Notably, higher incidence has been observed in tropical areas around the globe [3, 4].

Phlebotomines (Diptera: Psychodidae, Phlebotominae) are medically important insects popularly known as sand flies, involved in the transmission of bacteria, protozoan and arboviruses to humans and animals [5, 6]. At least 530 species of sand flies classified in 22 genera were already reported in the Americas [7]. Species belonging to genus *Lutzomyia* are highly anthropophilic, occur in a wide geographical distribution and constitute important vectors of human infections in the Americas.

Currently sand flies are associated with transmission of arboviruses belonging to the families *Rhabdoviridae* (genus *Vesiculovirus*), *Phenuiviridae* (genus *Phlebovirus*) and *Reoviridae* (genus *Orbivirus*). Sporadic reports of human cases indicate these infections are incidental in South America, associated mainly with recreational or occupational incursions into tropical sylvatic forests. These viruses are relatively common in Brazil, since at least 20 phleboviruses were discovered in the Brazilian Amazon region between 1954 and 1994 [5]. The lack of differential diagnostic methods and the common febrile symptoms may contribute to the underreporting of these infections.

Sand flies are commonly reported in Mato Grosso (MT) State, including in sylvatic areas of the Pantanal and Cerrado biomes [6, 8] and no viral diversity studies using metagenomics have been performed so far. For this reason, this study aimed to identify the presence of viruses in sand flies captured in North Pantanal and Chapada dos Guimarães National Park of MT.

## Methods

### Sampling of sand flies

Mato Grosso represents 35% of the Pantanal, the largest tropical humid territory worldwide, with warm and humid climate and mean annual precipitation between 800 and 1400 mm, 80% occur between November and March [9]. Chapada dos Guimarães National Park (CGNP) is a protected area of Cerrado presenting a dry winter from May to September and a rainy summer from October to April, with mean annual precipitation between 1800 and 2000 mm [10].

**Fig. 1:**
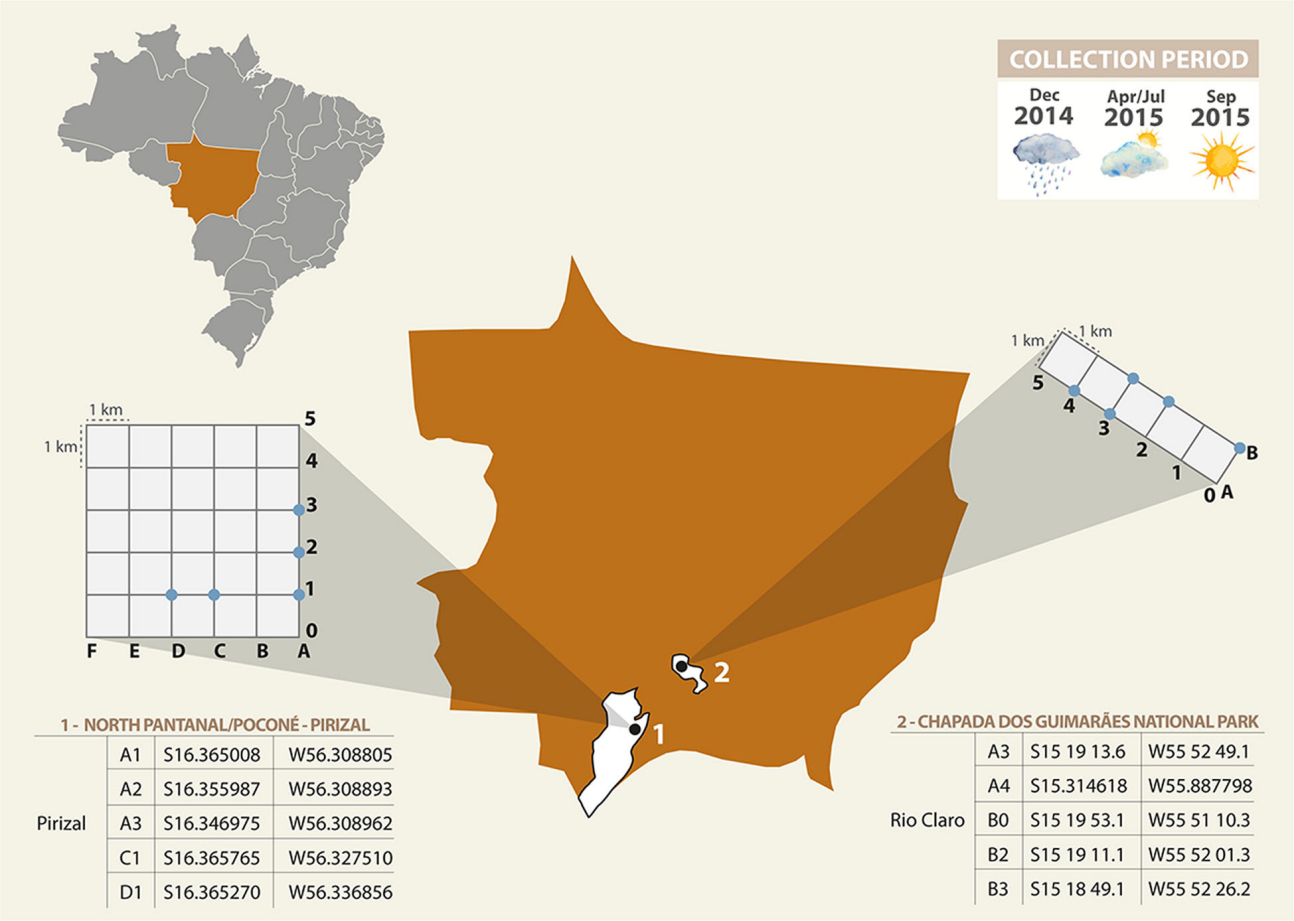
Location of RAPELD systems used in the study for sand flies collections in three climatic periods in North Pantanal (1) and Chapada dos Guimarães National Park (2), Mato Grosso State, Midwestern Brazil. Collection plots are marked with blue dots in each RAPELD system grade

Sand flies were captured in RAPELD grids (Rapid Assessment Program Long Term Ecological Research) at Pirizal, North Pantanal (16°14'06"S, 56°22'70"W) and at Rio Claro, CGNP (15°19'10"S, 55°52'13"W) (Fig. 1).

Five plots were selected in each location according to animal presence, proximity to water collections, access to vehicles, presence of riparian vegetation and habitat diversity.

Sand flies were captured with five CDC light traps placed along a 250 m transect at 50 m intervals between 18:00 h and 6:00 h at each sampling plot for two consecutive days in each climatic period. These specimens were immobilized for 1 min at -20 °C, identified according to dichotomous keys [11] and named [7]. Specimens were transported in liquid nitrogen to the virology laboratory. This study was approved by the System of Biodiversity Authorization and Information (SiSBio; ICMBio; IBAMA) under the number 43909-1.

### RNA extraction, random PCR and high throughput sequencing (HTS)

Pools of sand flies were macerated in phosphate saline buffer and centrifuged at 4 °C, 5,000× *g* for 4 min. The supernatant (0.2 ml) was subjected to RNA extraction with High Pure Viral RNA kit (Roche, Basel, Switzerland), without RNA carrier. RNA was then quantified with quantifluor RNA system (Quantus fluorometer, Promega, Madison, Wisconsin, USA).

Reverse transcription was performed with a mean of 152 ng of RNA, followed by cDNA double-stranded (dscDNA) synthesis and random PCR reactions in quintuplicate. PCR products were purified with 20% polyethylene glycol 8000 (20% PEG) and quantified with quantifluor one dsDNA system [12–14].

The library was prepared with TruSeq RNA Sample Prep v2 Kit (Illumina, San Diego, California, USA) (zx ≥ 100 ng of DNA product) followed by 2 × 100 paired-end sequencing in the Illumina HiSeq 2500 (Illumina), using two lanes and generating 60 GB.

### Sequence analysis and phylogeny

Sequences were analyzed with FastQC and trimmed (Trimmomatic 0.36) for the removal of primers sequences, adapters, and low-quality fragments (≤ 60 bp reads) removal (parameters: ILLUMINACLIP: TruSeq3-PE.fa:2:20:10, LEADING: 3, TRAILING: 3, SLIDINGWINDOW: 4:30, MINLEN: 60).

The assembled contigs, generated with different kmer (25, 40, 60 and 90) (Velvet v2.1.10), were compared with a viral RefSeq database from the National Center for Biotechnology Information (NCBI) using Blastx. Sequences with e-values > 1e-3 were compared to the entire nr database to exclude non-viral sequences.

The viral related contigs were manually inspected and annotated using Geneious R10. The length of some viral related contigs could be increased when the assembled reads extended beyond the contig ends. Therefore, reads were assembled back to the extended contig until the sequence could be extended no further.

The on-line open access software TMHMM (v2.0) (http://www.cbs.dtu.dk/services/TMHMM/) was used to predict transmembrane domains.

Viral sequences were deposited in the GenBank database under the accession numbers MF289182, MF289183 and MH119632. These sequences were aligned with other viral sequences using MAFFT (v7.221). The best evolutionary model for each dataset was determined with ProtTest (v2.4). Evolutionary history was inferred by maximum likelihood method and Jones-Taylor-Thornton (JTT) model with Gamma distribution with invariant sites (G+I). Phylogenetic trees were generated with MEGA7 and edited with FigTree (v1.4.3).

### Viral isolation in Vero cell culture, RT-PCR and transmission electron microscopy

A dilution (1:10 in 1 ml of inoculum) in RPMI medium of the pool supernatant positive for a phlebovirus was inoculated in a Vero cells (ATCC CCL-81) monolayer cultivated in T25 flasks for 2 h at 37 °C under constant agitation. After this period, RPMI culture medium with 5% fetal bovine serum was added and cells monitored daily for 5–7 days. Four passages were performed.

Supernatant was harvested for each passage and stored at -80 °C; monolayers were subjected to total RNA extraction (Trizol, Invitrogen, Carlsbad, Califórnia, USA) followed by a RT-PCR for a region of the L segment of Viola virus [primers SegLF (5'-AAG AGA CTC ATG GAC TCT GCT A-3') and SegLR (5'-CCG GGA AGT ATT TTC ATG GC-3')].

After reverse transcription (8 μl of RNA, primers at 2.5 μM) with 100 U of GoScript (Promega, Madison, Wisconsin, USA), a PCR containing the same primers at 1 μM, reaction buffer, MgCl_2_ (2 μM), DNTP mix (0.2 μM), 2.5 U of HotStart DNA polymerase (Promega, Madison, Wisconsin, USA) and ultrapure water in 50 μl reactions was amplified at 94 °C for 1 min, 30 cycles of 94 °C for 1 min, 56 °C for 1 min and 72 °C for 1 min, and a final extension of 72 °C for 2 min. PCR products (460 bp) were purified with 20% PEG and sequenced. At 5 days post-infection, the supernatant of Vero cells infected with Viola virus passage 5 was centrifuged at 159,000× *g* for 75 min using 3 ml of 25% sucrose cushion in polyallomer ultracentrifuge tubes (Beckman Coulter, Atlanta, Georgia, USA). The pellet was resuspended in PBS-1X and prepared for transmission electron microscopy (TEM) by negative staining [15] and observed in a JEOL JEM-1010 TEM at 100 kV.

## Results

### Specimens of sand flies captured in RAPELD systems at North Pantanal and Chapada dos Guimarães National Park

In total, 106 sand flies captured in the rainy (*n* = 86; 81.2%), transitional (*n* = 3; 2.8%) and dry (*n* = 17; 16.0%) periods, 38 (35.8%) in the CGNP and 68 (64.2%) at North Pantanal, comprised three pools of *Lutzomyia* sp., one of *Lutzomyia* (*Lutzomyia*) *longipalpis*, two of *Nyssomyia whitmani*, one of *Evandromyia* (*Aldamyia*) *evandroi,* one of *Ev.* (*Ald.*) *carmelinoi,* one of *Lu.* (*Tricholateralis*) *sherlocki*, one of *Ev.* (*Eva.*) *wilsoni* and two of *Brumptomyia* sp. (Table 1).

**Table 1:** Viral sequences obtained by high throughput sequencing from sand flies pools captured in the rainy, intermediate and dry period at North Pantanal and Chapada dos Guimarães National Park, Mato Grosso, Brazil

Place	Period	Pool	Species (*n*)	Reads	Contigs	Viral hits	Genome	Size (nt)	Identity (%)^a^	E value	GenBank ID
North Pantanal	Rainy	1	*Evandromyia* (*Aldamyia*) *evandroi* (5)	983,899,176	11,747	–	–	–	–	–	–
		2	*Lutzomyia* sp. (25)	849,154.470	61,862	–	–	–	–	–	–
		3	*Ev.* (*Eva.*) *wilsoni* (11)	819,202,516	43,993	–	–	–	–	–	–
		4	*Evandromyia* (*Aldamyia*) *carmelinoi* (1)	1,179,702,220	91,115	–	–	–	–	–	–
		5	*Lu.* (*Tricholateralis*) *sherlocki* (3)	449,698,864	28,893	–	–	–	–	–	–
		6	*Brumptomyia* sp. (15)	785,799,190	73,656	*Vesiculovirus*	ssRNA-	69	44.9	3.83e-12	–
	Transitional	7	*Lu. longipalpis* (3)	645,865,104	13,763	*Phlebovirus* (Seg L)	ssRNA-	6341	61	0	MF289183
						*Phlebovirus* (Seg M)	ssRNA-	4403	46	0	MF289182
						*Phlebovirus* (Seg S)	ssRNA-	1747	53	2e-79	MH119632
	Dry	8	*Lutzomyia* sp. (5)	1,115,860,120	94,199	*Vesiculovirus*	ssRNA-	72	40.3	4.10e-11	–
						*Flavivirus*	ssRNA+	48	47.9	5.88e-10	–
CGNP	Rainy	9	*Lutzomyia* sp*.* (22)	999,786,678	28,460	–	–	–	–	–	–
		10	*Nyssomyia whitmani* (3)	787,599,818	37,990	–	–	–	–	–	–
		11	*Brumptomyia* sp*.* (1)	–	–	–	–	–	–	–	–
	Dry	12	*Nyssomyia whitmani* (12)	692,307,934	30,666	–	–	–	–	–	–

^a^Identity refers to the closest phlebovirus species Chagres virus indicated by BLASTx (GenBank)

*Abbreviations*: *CGNP* Chapada dos Guimarães National Park, *n* number, *nt* nucleotides, *Seg* segment

Specimens of *Lu. longipalpis* were identified using the HTS data against a database of ND4 NADH dehydrogenase genes from eukaryotes. To avoid misidentification, only contigs > 300 nt were used in this analysis (data not shown).

### Sequencing data analysis

Illumina sequencing generated 9,308,876,090 reads and 516,344 contigs from 12 pools. BLASTx analysis of the contigs against a RefSeq viral database resulted in 1198(0.23%) viral hits.

A substantial amount of the assembled reads had no significant similarity to any of the sequences deposited in the viral RefSeq database. BLASTx (nr) generated hits with less than 100 bp with some viral families. Three pools of sand flies captured in North Pantanal in the three climatic periods presented contigs of 43–57 nt with 40–47% of identity with members of the families *Rhabdoviridae* (genus *Vesiculovirus*) and * Flaviviridae* (genus *Flavivirus*) (Table 1).

### Characterization of a new species of *Phlebovirus*

One pool containing extracts of three specimens of *Lu. longipalpis* females and males captured in the transitional period in Pirizal, North Pantanal presented the genome of a tripartite segmented virus belonging to phlebotomus fever serogroup, genus *Phlebovirus*, family *Phenuiviridae*, order *Bunyavirales*.

The partial genome sequence of this virus showed a low similarity with other species classified in the same genus and therefore, represents a putative new viral species that was named Viola phlebovirus.

**Fig. 2:**
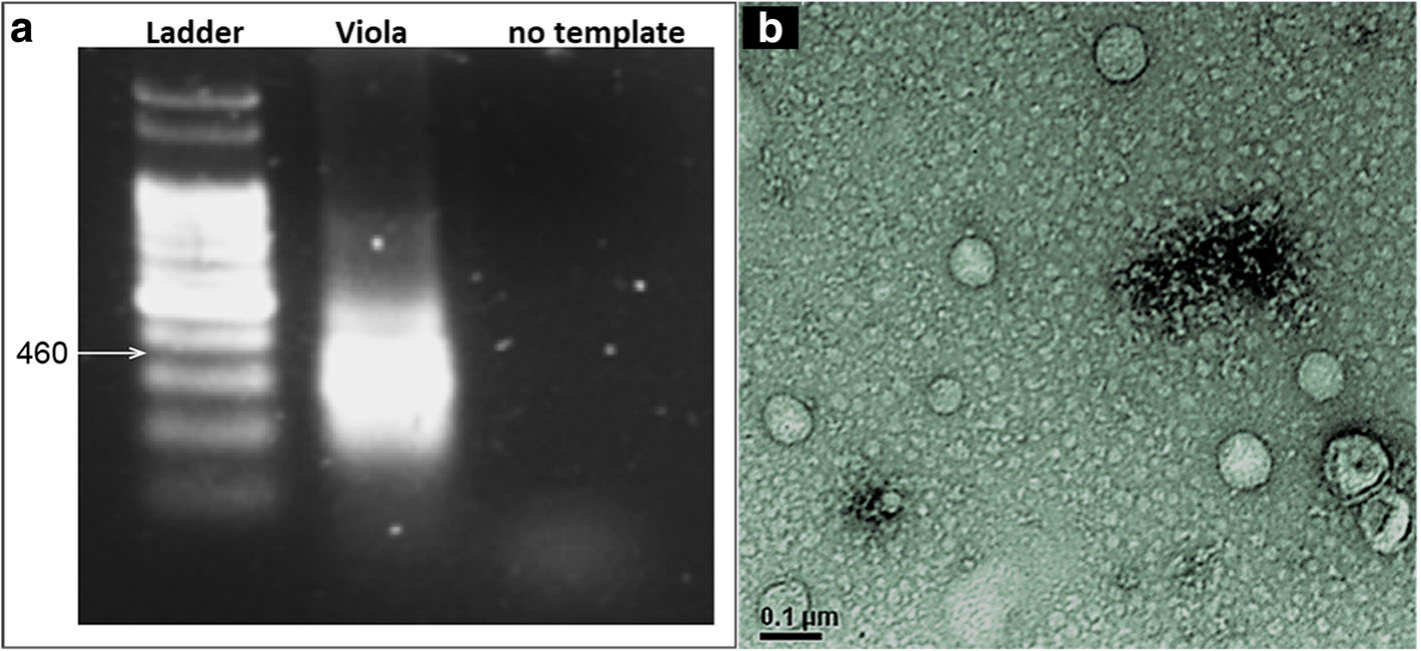
RT-PCR for a region of L segment (460 bp) at passage 4 (**a**) and transmission electron microscopy of Viola phlebovirus in Vero cells at passage 5 (**b**)

After the identification through HTS, this virus was isolated in Vero cells in the fourth passage and confirmed by RT-PCR using L segment specific oligonucleotides and nucleotide sequencing, and also by visualizing the viral particles in the supernatant after TEM (Fig. 2).

**Fig. 3:**
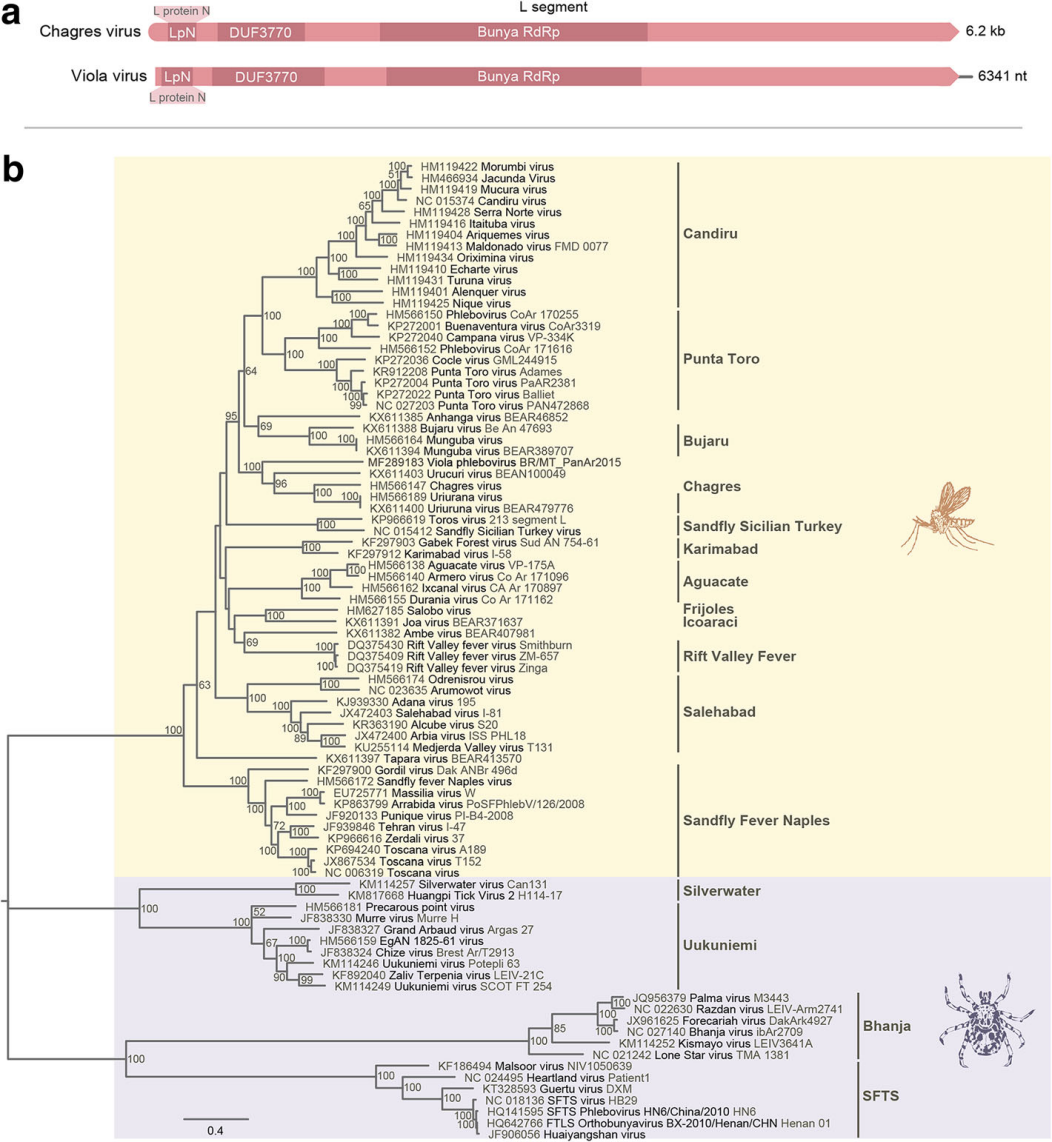
**a** Genomic organization of Viola phlebovirus L segment; and **b** phylogenetic tree based on amino acid sequences of phleboviruses by maximum likelihood method Jones-Taylor-Thornton model. Phlebotomus fever and Uukuniemi serogroups are marked in orange and purple, respectively. Bars indicate serocomplexes

The L segment presents 6341 nucleotides (nt) in length and encodes the RNA-dependent RNA polymerase (RdRp) (2082 amino acids, aa) (Fig. 3a). The main RdRp domain of Viola phlebovirus has 662 aa, the L protein N domain has 85 aa and the subdomain DUF 3770 has 297 aa. BLASTp analysis revealed 61% identity with the closest phlebovirus, Chagres virus.

**Fig. 4:**
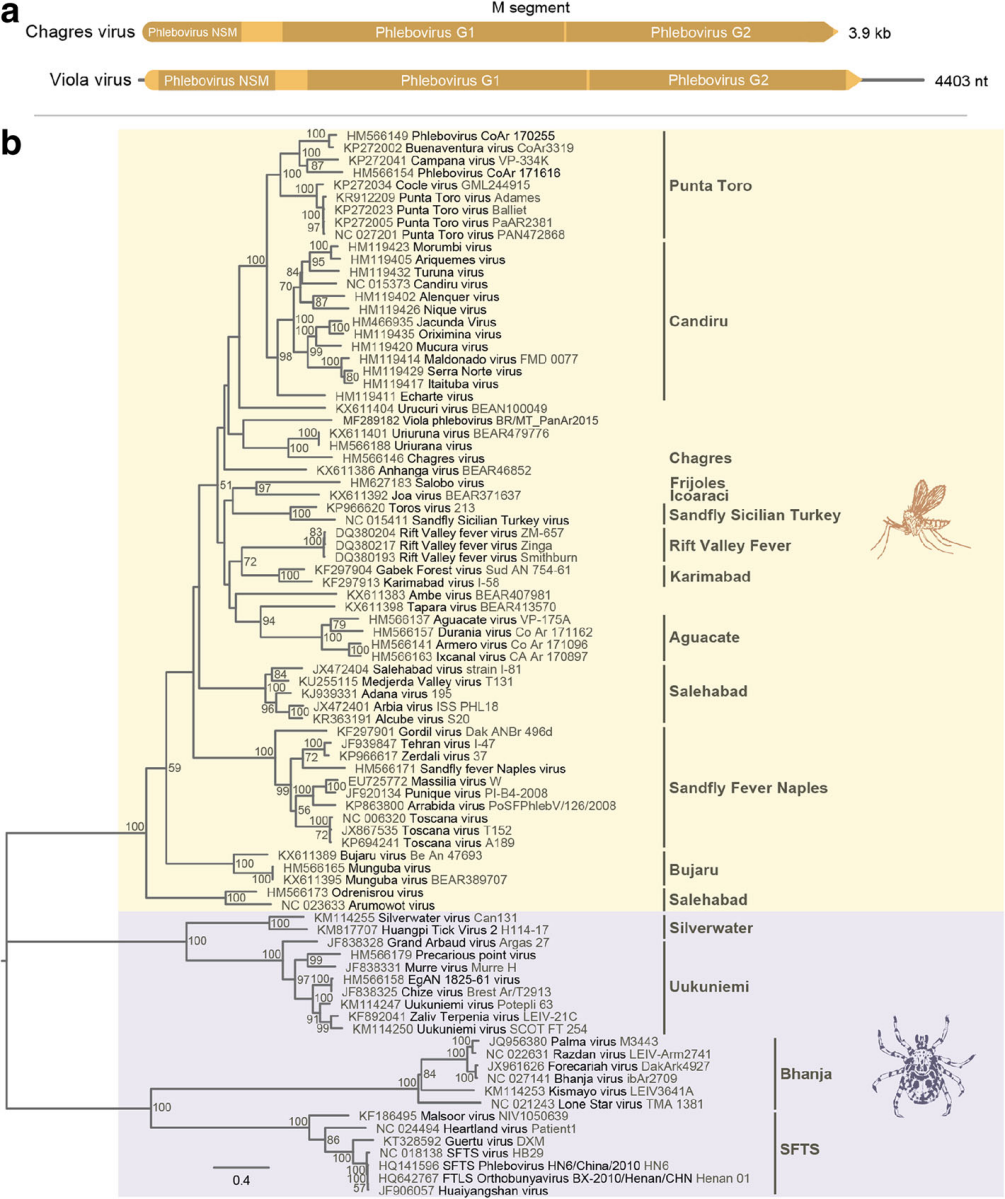
**a** Genomic organization of Viola phlebovirus M segment; and **b** phylogenetic tree based on amino acid sequences of phleboviruses by maximum likelihood method Jones-Taylor-Thornton model. Phlebotomus fever and Uukuniemi serogroups are marked in orange and purple, respectively. Bars indicate serocomplexes

The M segment is 4442 nt in length and encodes a polyprotein (4423 nt, 1467 aa) processed into: (i) NSm protein (41–259 aa; 219 aa of length), characteristic of phlebotomus fever serogroup; (ii) the G1 glycoprotein (315–840 aa; 526 aa of length); and (iii) the G2 glycoprotein (845–1324 aa; 480 aa of length) (Fig. 4a). Transmembrane domains were identified in the initial portion of G1 (741–763 aa), at the final region of G2 (1315–1337 aa) and a third between the end of G1 and beginning of G2 (828–850 aa). These domains are classical for phleboviruses, responsible for the anchoring of these glycoproteins to viral envelope [16]. BLASTp analysis revealed 43% of identity with the closest phlebovirus, Uriurana virus.

**Fig. 5:**
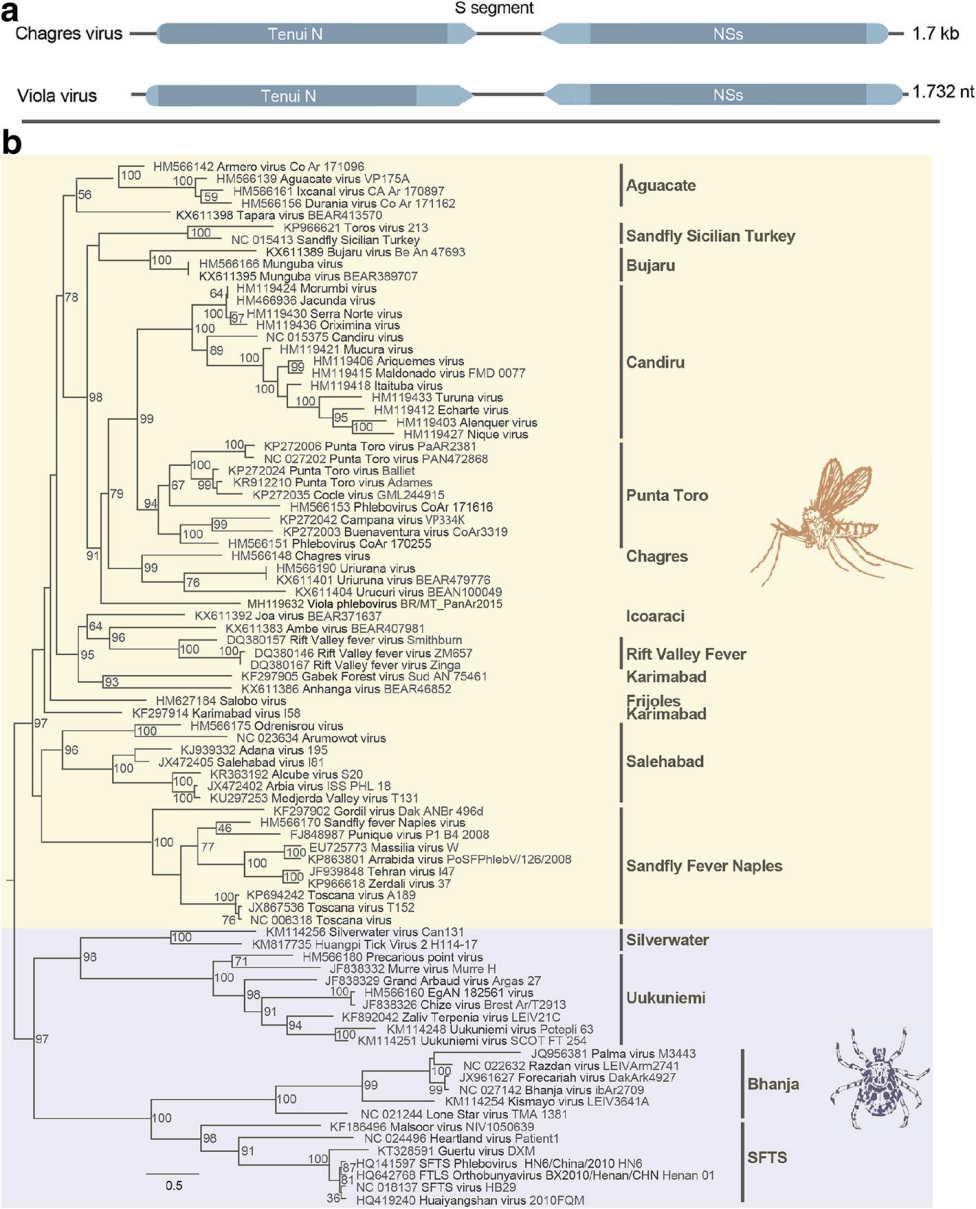
**a** Genomic organization of Viola phlebovirus S segment; and **b** phylogenetic tree based on amino acid sequences of phleboviruses by maximum likelihood method Jones-Taylor-Thornton model. Phlebotomus fever and Uukuniemi serogroup are marked in orange and purple, respectively. Bars indicate serocomplexes

The partial S segment is 1732 nt in length and encodes the nucleoprotein (N) and a nonstructural (NSs) protein in an ambisense coding strategy (Fig. 5a). The N protein (42–779 nt; 245 aa) presents the characteristic Tenui N domain of pheboviruses (pfam05733), involved in nucleocapsid formation. The NSs protein (1732–929 nt; 267 aa) codified by ambissense strategy presents the NSs domain (pfam11073), which is a major determinant of virulence by antagonizing interferon beta gene expression. BLASTp analysis revealed 49% and 37% identity (N and NSs, respectively) with the closest phlebovirus, Uriurana virus.

Phylogenetic analysis of the aligned amino acid sequence of the RdRp, NSm/glycoproteins and nucleoprotein suggested that Viola phlebovirus is a distinct member of the family *Phenuiviridae*, genus *Phlebovirus*, phlebotomus fever serogroup, clustering with Urucuri virus, Changres virus and Uriurana virus (Figs. 3b, 4b, 5b).

As previously shown, the pairwise comparisons of the RdRp and nucleoprotein (the two most conserved virus proteins) showed similarity of 61% and 49%, respectively (Additional file 1: Figure S1). Taken together, these results confirm that Viola phlebovirus is indeed a novel phlebovirus.

## Discussion

Viruses are the most diverse microorganisms. Metagenomic analysis has contributed to the description of viral diversity in several ecosystems and hosts, allowing the discovery of several previously unknown viral species and, allowing more precise and complete description of viral evolution. Despite the large number of arboviruses described in sand flies in the Amazon, studies involving viral detection in those arthropods are lacking in Midwestern Brazil. In this study, a new species of *Phlebovirus* belonging to phlebotomus fever serogroup was identified in *Lu. longipalpis* captured at North Pantanal.

In Brazil, sand flies have been associated with transmission of arboviruses only in the Amazon region [6, 17, 18]. Among these, the most relevant to public health are those belonging to the family *Phenuiviridae*, genus *Phlebovirus*, phlebotomus fever serogroup, associated with acute mild febrile illness in humans [19]. Despite their public health importance, genomic diversity of viruses associated with sand flies remains largely underestimated.

Previously, *Bunyaviridae* was a large family of negative or ambissense tripartite single stranded RNA viruses, primarily classified by serological methods. Recently, this family was reclassified into a new order, *Bunyavirales*, which contains now nine viral families, including *Phenuiviridae*, where the genus *Phlebovirus* was allocated [20]. Diversity and evolution of these viruses is largely associated to plasticity of their RNA genomic segments, resulting in point mutations, recombination and reassortment events [21]. These characteristics have contributed to the unfeasibility of serological tests to classify novel phleboviruses against all other known members of this viral order.

Within phleboviruses, S segment presents ambissense strategy, encoding two ORFs: the NSs protein associated with the ability of the virus to replicate in mammalian cells, and the N protein. M and L segments are negative sense segments encoding the envelope glycoproteins and NSm protein and the RdRp, respectively [22, 23]. Since the Uukuniemi serogroup does not encode NSm protein, the finding of NSm in the M segment of Viola phlebovirus indicates this virus belongs to phlebotomus fever serogroup, which includes arboviruses. NSm is a virulence factor associated with the inhibition of apoptosis in infected cells [24].

Viola virus was more closely related to phleboviruses from phlebotomus fever serogroup isolated from sand flies (Uriurana virus) and rodents *Proechimys guyannensis* (Urucuri virus) in the Amazon and in the Utinga Forest, State of Pará, respectively [25], and also isolated from humans with febrile illness in Panamá (Chagres virus) [26].

In addition, viral isolation of Viola virus in mammalian cell lines (Vero cells) and the presence of NSs and NSm proteins indicate that it is not an insect-specific virus (ISV) and might represent a virus that infect vertebrates.

## Conclusions

This detection and isolation of a putative new *Phlebovirus* (Viola phlebovirus) in *Lu. longipalpis* and its initial characterization contributes to our knowledge about viral diversity in arthropods from sylvatic areas of North Pantanal. Phylogeny revealed proximity with viruses causing disease in humans, rodents and isolated from sand flies belonging to phlebotomus fever serogroup. Isolation of Viola virus in mammalian cells indicates this virus is not an ISV and represents a novel species with no known vertebrate host. Therefore, future studies involving Viola phlebovirus may address its importance for public or veterinary health. This is the first study reporting a new species of virus infecting arthropods through HTS and able to replicate in Vero cells in Mato Grosso, Midwestern Brazil.

## Additional file

Additional file 1: **Figure S1.** Pairwise amino acid comparision of Viola phlebovirus M segment sequences with all other M segment sequences of  members of the genus *Phlebovirus* available in the GenBank database. (PNG 62 kb)

## Abbreviations

HTS: high throughput sequencing
TEM: transmission electron microscopy
RNA: ribonucleic acid
RdRp: RNA dependent RNA polymerase
RT-PCR: reverse transcription polymerase chain reaction
RAPELD: Rapid Inventary Long Term Ecological Research
aa: amino acid
G: glycoprotein
L: large
M: medium
S: small
MT: Mato Grosso
CGNP: Chapada dos Guimarães National Park
CDC: Centers for Diseases Control and Prevention
NCBI: National Center for Biotechnology Information
cDNA: complementary deoxyribonucleic acid
dscDNA: double-stranded cDNA
SegLF/SegLR: segment L forward and reverse primers
SiSBio: System of Biodiversity Authorization and Information
ICMBio: Chico Mendes Institute for the Conservation of Biodiversity
IBAMA: Brazilian Institute of the Environment and Renewable Natural Resources
PEG: polyethyleneglycol
BLAST: basic local alignment search tool
RefSeq: reference sequence
Ev/Eva: *Evandromyia*
Ald: *Aldamyia*
Lu.: *Lutzomyia*; nt: nucleotide
nr: non-redundant
N: nucleoprotein
ORF: open reading frame
ISV: insect-specific virus
