# Supplementary figures and images for: Viola phlebovirus is a novel Phlebotomus fever serogroup member identified in Lutzomyia (Lutzomyia) longipalpis from Brazilian Pantanal
Source: Parasit Vectors. 2018 Jul 11;11:405. doi: 10.1186/s13071-018-2985-3 (PMC6042282; doi:10.1186/s13071-018-2985-3)

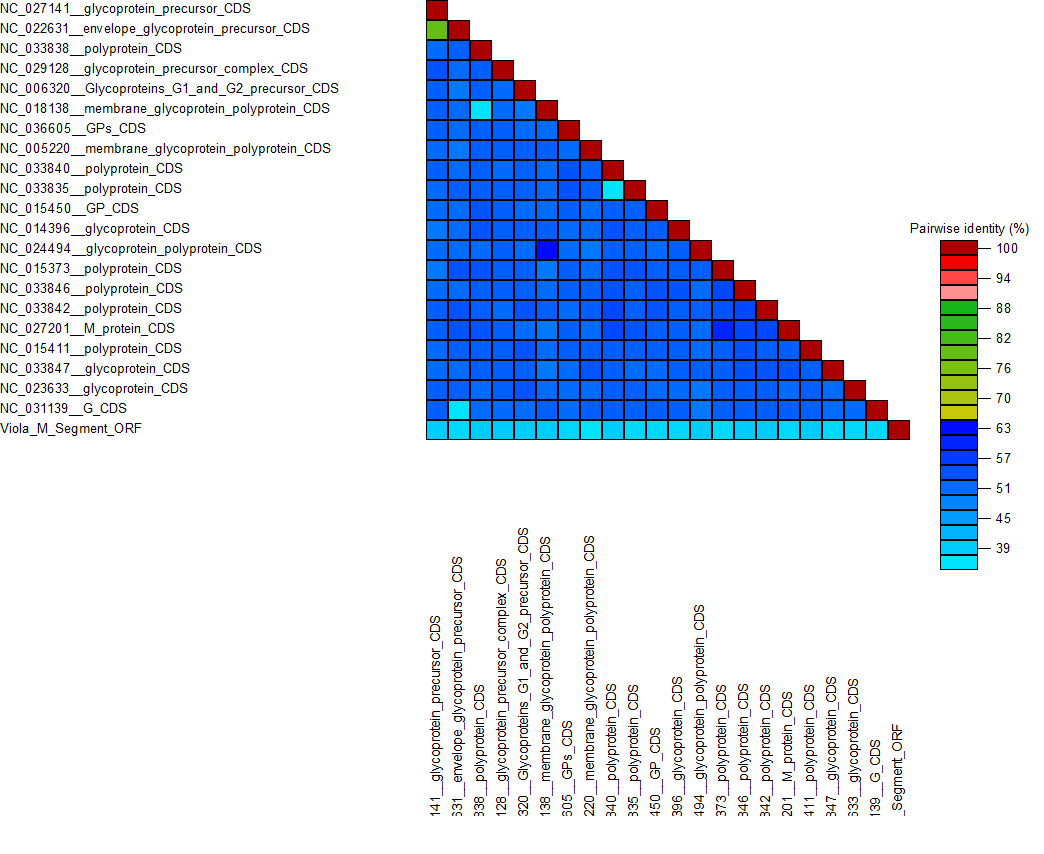

Supplement: Supplementary file 1 — Figure S1. Pairwise amino acid comparision of Viola phlebovirus M segment sequences with all other M segment sequences of members of the genus Phlebovirus available in the GenBank database. (PNG 62 kb) [file 13071_2018_2985_MOESM1_ESM.png]
